# Orthorexia Nervosa: Disorder or Not? Opinions of Dutch Health Professionals

**DOI:** 10.3389/fpsyg.2019.00555

**Published:** 2019-03-15

**Authors:** Frida V. M. Ryman, Tomris Cesuroglu, Zarah M. Bood, Elena V Syurina

**Affiliations:** Athena Institute, Faculty of Science, Vrije Universiteit Amsterdam, Amsterdam, Netherlands

**Keywords:** orthorexia nervosa, eating disorder, diagnosis, health professionals, categorization, classification

## Abstract

**Introduction:** Orthorexia nervosa (ON) is a condition that is characterized by a pathological obsession with eating foods one considers healthy and has recently been suggested as a new possible diagnosis. However, there is limited published research on health professionals’ recognition, ideas and opinions regarding the diagnosis and classification of ON.

**Purpose:** The aim of this mixed-methods study was to gain insight into the perspectives of clinically active health professionals on ON, and into their opinions on if and how the disorder should be classified.

**Results:** Psychologists, psychiatrists, dietitians and physiotherapists in the Netherlands (*n* = 160) participated by responding to a self-administered questionnaire. Most health professionals (78%) reported that they thought that ON should have its own diagnosis. This opinion was more common in physical health professionals than in mental health professionals. A majority (74%) agreed that ON fits within the Diagnostic and Statistical Manual of Mental Disorders (DSM) category Eating and Feeding Disorders. Interviews with 15 mental health professionals were analyzed using code frequencies and continuous comparisons. Mental health professionals reported believing that ON is prevalent in the general population and that a separate diagnosis would have both advantages and disadvantages for health professionals and patients. Interview participants described the typical ON patient as being young, female, and highly educated; characteristics that overlap with typical anorexia nervosa and obsessive compulsive disorder cases.

**Conclusion:** The results suggest that some health professionals from a heterogenous sample in the Netherlands think ON should have a separate diagnosis in the DSM, however, the study needs to be replicated to allow for further generalization. Methodological design of this study may be utilized in future research with similar aims. The findings can serve as a foundation for investigation of individuals’ experiences of distress caused by ON, and further refinement of the diagnostic criteria.

## Introduction

Eating disorders are serious and potentially fatal health problems that constitute a considerable burden of mental health problems (American Psychiatric Association, 2013; Institute for Health Metrics and Evaluation [IHME], 2015). However, disordered eating patterns are continuously changing as societal influences and pressures evolve. One of the latest eating patterns that have entered the discussion regarding eating disorders is orthorexia nervosa (ON). First mentioned in 1997 in a yoga journal by Bratman (1997), Dunn et al. (2017), orthorexia literally means proper or correct appetite and is characterized by a pathological obsession with eating foods one considers healthy.

Despite an increasing number of studies about ON, the body of scientific evidence is still highly fragmented. At present, ON does not have its own classification in the Diagnostic and Statistical Manual of Mental Disorders (DSM)-5, which is partially due to an ongoing discussion about how to properly classify ON (Vandereycken, 2011). According to some sources, ON shares features with other conditions, such as obsessive compulsive disorder (OCD) and anorexia nervosa (AN) (Koven and Abry, 2015). Other research suggests that ON may be best diagnosed as avoidant/restrictive food-intake disorder (ARFID); an eating or feeding disturbance (e.g., apparent lack of interest in eating or food, avoidance based on the sensory characteristics of food, concern about aversive consequences of eating), resulting in persistent failure to meet appropriate nutritional and/or energy needs (Kreipe and Palomaki, 2012; Dunn and Bratman, 2016). However, limited scientific research on ON means these classifications are based solely on the opinions and perceptions of the authors of these articles.

The controversy regarding the underlying pathology of the disorder and the lack of research on the subject have resulted in an absence of validated diagnostic criteria. Dunn and Bratman (2016) have proposed the following diagnostic criteria: A. an obsessive focus on “healthy” eating and emotional distress related to food choices that are perceived as unhealthy (evidenced by a number of further specified points) and B. clinical impairment due to the behavior and pre-occupation (evidenced by a number of further specified points). These criteria and the ORTO-15 are the most well-known instruments for diagnosing ON (Donini et al., 2004; Dunn and Bratman, 2016).

While attempts to determine the prevalence of ON have been made, the lack of consensus on diagnostic criteria for the condition means there is not yet a reliable estimate available. The available estimates of prevalence of ON range from less than 1 to 88.7%, with studies conducted in different population groups (Bosi et al., 2007; Fidan et al., 2010; Valera et al., 2014; Segura-Garcia et al., 2015; Dunn and Bratman, 2016). This range is obviously extremely wide, and no study has yet been conducted which has provided a reliable estimate.

Additionally the knowledge on practicing health professionals’ opinions regarding ON is lacking. This mixed methods study was conducted in the Netherlands (Spring-Summer 2018) in an attempt to fill this knowledge gap and move one step closer to understanding the phenomenon of ON. To the best of our knowledge, this study is the first to focus on the opinions and ideas of health professionals with regards to Orthorexia Nervosa.

The objective of the study was to assess the level of recognition of ON as a distinct disorder amongst health providers in the Netherlands and to uncover their opinions on the pathology, classification, and diagnostic criteria for the condition. Given that different health professionals have different experience and knowledge, their opinions and ideas on a specific topic may vary. For this reason, a distinction has been made between mental health professionals (MHP) (including psychiatrists and psychologists) and physical health professionals (PHP) (dietitians and physiotherapists).

## Materials and Methods

A mixed-methods design with concurrent triangulation was adopted, unifying the results from a self-administered online questionnaire and semi-structured interviews. This design was believed to provide the most insight on different health professionals’ opinions, as well as a deeper understanding of these in a smaller group of MHPs.

### Procedure

#### Questionnaire

We designed our questionnaire in order to collect initial data on the opinions of health professionals about ON in the Netherlands.

As no validated measures were available for the data collection required for this opinion study and no similar studies had been previously conducted, the co-authors created a questionnaire based on a framework that visualizes factors possibly influencing a health professional’s opinions regarding ON (Appendix A). The questionnaire was designed and first written in English by FR, ZB, and ES, as this was the language used within the research group (due to the multi-national nature of the group), and then translated to Dutch by ZB. Following the initial design, the relevance and formulation of the questions were discussed with a Dutch psychologist. Qualtrics Survey Software was used, and the questionnaire was launched in March 2017.

The questionnaire was digitally distributed among psychologists, psychiatrists, dietitians and physiotherapists (total *N* on the list: 1165). Inclusion criteria for participating in the survey were being in possession of a higher professional education or university degree (or equivalent) and being clinically active in the Netherlands.

The providers were then contacted via email and through social media and were presented with an anonymous link to the questionnaire. They were also asked to forward the questionnaire link to their colleagues.

##### Questionnaire lay-out

The questionnaire consisted of three sections: recognition, diagnosis and classification of ON; potential influence of modern Western culture on ON; and demographic characteristics of the respondents. Questions within the section on recognition, diagnosis and classification of ON included items such as whether participants recognized the described pattern of symptoms, what their subjective estimation of the prevalence of the disorder was, whether they found current diagnostic criteria sufficient and opinions about ON classification. Results regarding the potential influence of the modern Western culture can be found in another article published in this journal (Syurina et al., 2018) and will not be discussed in this paper. The questionnaire can be found in Appendix B.

#### Interviews

Semi-structured interviews were conducted to triangulate and enrich the quantitative data. An interview guide (Appendix C) was created and piloted in English. The process consisted of three separate interviews. All interviewers were present during these interviews and the interview guide was discussed and refined after each interview. The interview guide was translated to Dutch for interviews with Dutch speaking participants.

Clinically active psychologists and psychiatrists in the Netherlands were eligible to participate in the interviews if they had handled at least one eating disorder case in the last year. The list of health professionals used for recruitment of questionnaire participants was also used for recruitment of interview participants. Due to privacy protection we could not check for any overlap, i.e., whether interview participants were also the ones filling in the questionnaire. The recruitment occurred via email and telephone calls.

The participants signed an informed consent prior to the interviews. The interviews were then recorded and transcribed verbatim.

##### Interview structure

The interviews were semi-structured in nature and consisted of four parts: opinions on diagnosis and categorization, a hypothetical case based on current proposed criteria for ON, potential link to the modern Western culture and demographic information. The potential link to the modern Western culture has been discussed elsewhere (Syurina et al., 2018).

When discussing the hypothetical case, participants were presented with an infographic containing a figure surrounded by text boxes with symptoms of ON (Appendix D). Participants were asked to describe what kind of individual they envisioned when looking at this and how they would diagnose this patient.

### Participants

#### Questionnaire

A sample size calculation revealed that a total number of 88 participants was required to yield a power of 0.8 at alpha-level 0.05 with a Pearson’s *r* of 0.3 for the main outcome (a two-group comparison on a dichotomous variable). A total number of 160 participants (13.7% of the contacted clinicians) was reached. Eleven of these fell in the profession category “other,” where they defined themselves as e.g., associate professors, nutritionists, and psychotherapists. Two participants did not report their profession. 41 psychologists, 3 psychiatrists, 71 dietitians, and 34 physiotherapists were included in the study. They were primarily female with 44 MHP and 104 PHP participating (see Table 1).

**Table 1:** Questionnaire participant characteristics.

*Variable*	*N*	*%*
**Sex**		
Male	20	12.5
Female	138	86.3
Not listed	1	0.6
**Age**		
25 and younger	10	6.3
26 to 35	43	26.9
36 to 45	28	17.5
46 to 55	39	24.4
56 to 65	33	20.6
66 and older	2	1.3
**Highest level of education**		
Higher Professional Education	75	46.9
Bachelor	10	6.3
Master	61	38.1
Other ^a^	12	7.5
**Profession**		
Psychologist	41	25.6
Psychiatrist	3	1.9
Dietitian	71	44.4
Physiotherapist	34	21.3
Other^b^	9	5.6
**Years of experience**		
0 to 7	56	35
8 to 15	30	18.8
16 to 23	21	13.1
24 to 31	25	15.6
32 to 39	21	13.1
40 or more	1	0.6
**Encountered patient(s) with suspected or confirmed eating disorder**		
Yes	138	86.3
No	20	12.5
**Encountered patient(s) who fulfill ON criteria**		
Yes, within the last year	76	47.5
Yes, more than one year ago	25	15.6
No	59	36.9
Total *N*	160	100.0


*^*a*^ Includes e.g., pre-master, Ph.D, and higher professional education master.*

*^*b*^ Includes e.g., associate professors, nutritionists, and psychotherapists.*

#### Interviews

Fifteen individuals participated in the interviews: thirteen psychologists and two psychiatrists. Four participants were expatriates (origins were Norway, Romania, Turkey, and Greece), with the remaining eleven being native Dutch. The sample consisted of fourteen women and one man. Eleven had undertaken a master’s program, ten of whom had already or were in the process of further specializing. Work experience ranged from 3 to 32 years. Twelve of the individuals regularly met patients with eating disorders, while three participants only rarely encountered this patient group (see Table 2).

**Table 2:** Interview participant characteristics.

*Variables*	*N*	*%*
**Sex**		
Male	1	6.7
Female	14	90.3
**Age**		
25 and younger	0	0.0
26 to 35	2	13.3
36 to 45	5	33.3
46 to 55	4	26.7
56 to 65	4	26.7
66 and older	0	0.0
**Profession**		
Psychologist	13	86.7
Psychiatrist	2	13.3
**Origin**		
Dutch	11	73.3
Non-Dutch	4	26.7
**Education level above bachelor**		
Master	11	73.3
Doctoral	6	40.0
Specialization	10	66.7
**Study place**		
The Netherlands	9	60.0
Other	3	20.0
**Years of experience**		
0 to 7	2	13.3
8 to 15	7	46.7
16 to 23	3	20.0
24 to 31	2	13.3
32 to 39	1	6.7
40 or more	0	0.0
Regular contact with patient(s) with an eating disorder	12	80.0
Total *N*	15	100.0


### Data Analysis

#### Questionnaire

Statistical analysis was carried out in IBM SPSS Statistics Version 24. Binomial tests were conducted to test whether a significant proportion of the entire sample thought that ON should have its own diagnosis or whether they thought that exercise related symptoms should be part of the diagnostic criteria. The null-hypothesis in these tests was that the yes/no-groups would be of the same size. Between-group differences were analyzed using Chi-squared tests, with Fisher’s exact test being applied where less than 80% of the expected frequencies exceeded 5. The professional category “other” was excluded in the comparative analyses. All tests were two-tailed, and significance level was set at an alpha of 0.05. To calculate the achieved power, a *post hoc* power analysis was performed, set at alpha-level 0.05 and with a Pearson’s *r* of 0.3.

A *post hoc* power analysis revealed that a power of 1.0 was achieved for the main outcomes (Binomial tests, single sample) and 0.97 for the two-group comparisons (MHP and PHP). In the four-group comparisons (for separate professions) a power of 0.65 (for the question of what currently existing diagnosis ON would fit within) and 0.76 (for the question of what category ON falls within) was achieved.

#### Interviews

Qualitative analysis was carried out in ATLAS.ti 7. Thematic analysis with open and closed coding was applied. A code book (Appendix E) was created by FR and was based on the research question as well as emerging codes within interviews. Common themes were found and codes were continuously revisited and distributed throughout the analysis. FR coded all English transcripts and a native Dutch speaker aided in the coding of the Dutch interviews in order to ensure understanding and correct coding. Analysis was then carried out by FR by looking at frequencies of codes with continuous comparison with participant characteristics, looking for patterns between, for example, type of profession and responses.

### Ethics Statement

According to Dutch legislation, no ethical approval was required for this study (Wet medisch-wetenschappelijk onderzoek met mensen, 2018). Participation in the study was voluntary and participants were free to withdraw at any time. Interview participants signed an informed consent prior to participation and confidentiality of the collected data was ensured, as the data were stored on a password protected database. All participants were offered to the opportunity to be sent the results of the study.

## Results

Following the triangulated, mixed methods nature of the study, the results of qualitative and quantitative analysis are presented together and divided in four themes. First, the beliefs about relative prevalence of ON in Dutch population and level of recognition of ON in the daily practice will be presented. Next, opinions about how ON can be categorized within the framework of DMS will be discussed, followed by the views on a potential need for a separate diagnosis for ON. Finally, the impressions of the current proposed diagnostic criteria for ON will be presented.

### Recognition in Daily Practice

#### Questionnaire

In total, 95.6% reported believing that ON is, at least to some extent, prevalent in the general population in the Netherlands. None of the respondents thought that ON is highly prevalent in the Netherlands and seven participants (4.4%) stated believing that ON is not prevalent at all. There was no significant association between opinion about prevalence and professional group (*p* = 0.126). A majority of respondents (63.1%) reported having assisted clients who fulfilled the proposed diagnostic criteria (47.5% within the last year and 15.6% more than a year ago). PHP reported having handled more clients fulfilling the criteria for ON than did MHP (*p* < 0.001), however, no significant difference was found between specific professions (*p =* 0.072).

#### Interviews

The results of the questionnaire were supported by the interview data. Participants generally reported believing that ON is prevalent in the general population and recognized the condition in their daily practice as well as in their personal lives. They mentioned how healthy eating and caring about what one eats is becoming more and more prominent, with individuals looking not only to nutritional value, but also to often overlooked information about the quality of the food e.g., content of e-numbers (codes for substances that are permitted to be used as food additives) etc., (Table 5, quote 1), and experiencing an excessive need for control over food intake. Less common, yet mentioned by at least two participants, were obsessive compulsiveness, sportiness, and awareness (of the patient’s behavior not being good for them) (Table 5, quotes 2–4).

### Categorization

#### Questionnaire

Regarding categorization, the majority of questionnaire respondents reported believing that ON falls under the Eating and Feeding Disorders category in the DSM, followed by the category Obsessive-Compulsive and Related Disorders. Approximately a quarter said that they believed it could fit within Anxiety Disorders. Additional categories mentioned were Autism Spectrum Disorders and Personality Disorders (Table 3). The different professional groups (mental health vs. physical health professionals) were not significantly associated with categorization (*p* = 0.985), however, the separate professions were (*p* < 0.001). Physiotherapists favored the category Obsessive-Compulsive and Related Disorders (84.8%), with only 48.5% choosing the category Eating and Feeding Disorders, while individuals with other professions favored the Eating and Feeding Disorders category (75% or more), followed by the Obsessive-Compulsive and Related Disorders category (approximately 50%).

**Table 3:** Questionnaire frequencies.

*Variables*	*N*	*%*
**Estimated level of prevalence of ON in the general population^a^**		
1	7	4.4
2	84	52.5
3	63	39.4
4	6	3.8
5	0	0.0
**Separate diagnosis for ON**	124	77.5
**Diagnosis ON fits within^b,c^**		
Anorexia Nervosa	10	29.4
Bulimia Nervosa	2	5.9
ARFID	18	52.9
OCD	18	52.9
Generalized Anxiety Disorder	4	11.8
Other	4	11.8
**DSM category ON fits within^c^**		
Eating and Feeding Disorders	118	74.2
Obsessive-Compulsive and Related Disorders	89	56
Anxiety Disorders	38	23.9
Other	8	5.0
In favor of including exercise related symptoms in diagnostic criteria	110	68.8
Total *N*	160	100.0


*^*a*^ 1 = Not prevalent; 5 = Extremely prevalent.*

*^*b*^ Responses exclusively from participants opposed to a separate diagnosis. Percentages represent proportions of this group.*

*^*c*^ Multiple responses were possible.*

#### Interviews

Although interview participants reported believing that ON fits within several categories of the DSM, their responses were consistent with the questionnaire participants’, in that a clear majority (*n* = 11) favored the category Eating and Feeding Disorders. Two other categories were mentioned as preferred categories: Obsessive-Compulsive and Related Disorders and Anxiety Disorders (Table 5, quotes 5 and **6**). Though not preferred over Eating and Feeding Disorders, two participants also mentioned the category Autism Spectrum Disorders (Table 5, quote 7).

### Separate Diagnosis

#### Questionnaire

As shown in Tables 3, 4 most questionnaire participants agreed that ON should have a separate diagnosis (*p* < 0.001). A majority of those in opposition to this opinion thought that ON fit within the conditions ARFID and/or OCD. As depicted in Table 4, significant differences were found between the different profession groups (*p* = 0.003) regarding opinions on giving ON its own diagnosis, with PHP favoring a separate diagnosis to a greater extent than MHP (84.8% compared to 67.4%). Psychiatrists were consistently opposed to a separate diagnosis.

**Table 4:** Statistical testing.

*Variables*	*Test statistic*	*P-value*
Separate diagnosis for ON	Binomial test	<0.001^∗^
Profession group x Separate diagnosis for ON	χ2 test	= 0.003^∗^
Profession x Separate diagnosis for ON	Fisher’s exact test	= 0.003^∗^
Level of education x Separate diagnosis for ON	Fisher’s exact test	= 0.117
Profession group x Diagnosis ON fits within	χ2 test	= 0.112
Profession x Diagnosis ON fits within	χ2 test	= 0.432
Level of education x Diagnosis ON fits within	χ2 test	= 0.067
Profession group x Category ON fits within	χ2 test	= 0.985
Profession x Category ON fits within	χ2 test	<0.001^∗^
Level of education x Category ON fits within	χ2 test	= 0.267
Exercise related symptoms in diagnostic criteria	Binomial test	<0.001^∗^
Profession group x Exercise related symptoms in diagnostic criteria	χ2 test	= 0.971
Profession x Exercise related symptoms in diagnostic criteria	Fisher’s exact test	= 0.373
Level of education x Exercise related symptoms in diagnostic criteria	Fisher’s exact test	= 0.740


*^∗^Significant at 0.05 level.*

#### Interviews

Regarding giving ON its own diagnosis, the interview participants were uncertain. Approximately half of the participants (*n* = 7) clearly stated that they were in favor, however, only one was clearly opposed, thinking that it was a subcategory of AN (Table 5, quote 8). One participant mentioned that ON was not likely to appear in the upcoming versions of the DSM, due to the long and complex process of incorporating new diagnoses (Table 5, quote 9).

**Table 5:** Participant quotes.

Reference	Quote	Reference	Quote
**1**	Expatriate psychologist: *[...] considering the flow in the work now, it is becoming a more prominent issue I believe, because I realize in my personal life and I am encountering with other people deeply, there is an issue like this [...] toxicity in eating or eating organic food or like purification of the body becoming a really kind of health-related content for all the talks around me.*	**11**	Expatriate psychologist: *I would go for eating disorders not otherwise specified. Because, for now, for what I know already, and because this is so brand new. It feels like, more, the safe side. Because then, if it’s an eating disorder, which seems to be somehow an eating situation here … At least you’re under the relevant category.*
**2**	Expatriate psychologist: *Young, highly educated woman I have with my experience in mind.*	**12**	Expatriate psychologist: [...] *the more training programs we have […] if we have a diagnosis for it we can have a facility for it and we can have specialists for it.*
**3**	Dutch psychologist: *Perfectionist, rigid, in need for control.*	**13**	Dutch psychologist: *[...] she (the patient) had to be hospitalized, which had to be done through her health insurance and otherwise her treatment would not have been reimbursed. [...]She does suffer from what we call Orthorexia, but that is not included (in the DSM) [...] so in terms of that her diagnosis will be adapted.*
**4**	Expatriate psychologist: *So they’re pretty aware, actually, of different things. And can also know, that it’s imbalanced, what they’re doing, but they’re just not able to stop it.*	**14**	Expatriate psychologist: *And it’s an advantage for us as […] psychologists, that work with treating it. To be able to communicate and convey it in a different way, more clear.*
**5**	Expatriate psychologist: *Now because the control is really really important […] obsessive-compulsive disorder.*	**15**	Expatriate psychologist: [...] o*ur current diagnostic statistical manual is updated all the time with what happens around. And [...] it would give, like, the scientific community an idea that, okay, we are, we are not an outdated system here, but we really, we really listen to what happens around, and we try to incorporate that, in the way we work.*
**6**	Dutch psychologist: *I would now be inclined to classify it under Anxiety Disorders, possibly under OCD and related disorders.*	**16**	Dutch psychologist: *Yes a disadvantage could be, but to be honest I don’t really think so, that a vast amount of diagnoses arise. You could also say that [...] this is covered by restricting type of eating disorder. [...] Then you don’t get a jumble of diagnoses.*
**7**	Dutch psychologist: *It could also fit with someone with autism who has a rigid idea about what’s healthy food and can’t think flexible anymore.*	**17**	Dutch psychologist: *If you call everyone who stops eating gluten orthorexic, then we have a problem.*
**8**	Dutch psychiatrist: *[…] anorexia nervosa in the DSM also includes severity categories, and a mild form of Anorexia Nervosa really resembles this.*	**18**	Expatriate psychologist: *So if this social influence we get on eating healthily and all that, comes from a social influence perspective. By highlighting, not that, but highlighting the people who have the disorder, it’s more distracting everyone from what is the actual root of the problem.*
**9**	Dutch psychologist: *See when you look at the process of determining a new classification [...] that is a very lengthy process, and the outcomes of that process have to meet certain criteria.*	**19**	Expatriate psychologist: *Normally dsm is very specific. […] Compulsive behavior is very good, and maybe more examples would be needed here. Normally dsm gives examples on those.*
**10**	Expatriate psychologist: *No, I would use this one (ON diagnosis) for sure, because this can help, also, the patient to see more clear, in which way this is impairing their everyday life. Quality of well-being actually.*	**20**	Dutch psychologist: *I find the medical complications somewhat general. [...] It is described in anorexia [...]. And things like loss of hair, and yeah, disturbed menstruation, stomach and bowel issues, that kind of stuff. So it could be a bit more extensive.*


When discussing a hypothetical case, further opinions about separate diagnosis for ON were expressed. Despite only seven participants being outspokenly in favor of a separate diagnosis for ON, eleven of the fifteen participants reported that they would use the new diagnosis rather than the currently available ones for the hypothetical case, had it been an option. Only four participants would continue with the currently available ones, and two of these reported that they would do so because it is “safer” – because they feel more secure with the current ones due to them being more established (Table 5, quote 10 and 11).

Regardless of favoring or opposing a separate diagnosis, interview participants reported several advantages and disadvantages of introduction of ON as a diagnosable disorder.

##### Advantages of a separate diagnosis

Two large themes in prospective advantages of ON diagnosis arose: improvements for patients, and improvements for health professionals. The participants reported that a separate diagnosis would result in more research on the condition, more programs to learn about it and specialize within it, and more facilities to treat this specific condition; thereby leading to better treatment (Table 5, quote 12). It was also mentioned that treatment guidelines may then be developed. Other improvements for patients included that having a diagnosis or label might help some patients cope with their condition, and that insurance reimbursement would then be possible for patients with ON. Presently, health professionals sometimes find themselves in situations where they have to set a diagnosis that is not in complete concordance with the symptoms, in order to warrant reimbursement (Table 5, quote 13).

Potential improvements for health professionals included helping them to talk about the condition with their clients and knowing which direction to look in, as well as the creation of guidelines to help practitioners to treat this patient group. Keeping the DSM up-to-date with societal changes was also mentioned (Table 5, quotes 14 and 15). Finally, four of the participants mentioned that there are “layers” to eating disorders, whereby eating disorders in reality are more nuanced than what current diagnoses suggest. With this in mind, a separate diagnosis for ON might help to display this and develop the concept of eating disorders further.

##### Disadvantages of a separate diagnosis

Regarding disadvantages of a separate diagnosis, no differentiation between disadvantages for patients and for health professionals was clear. The main disadvantage reported in the interviews was that there are already too many diagnoses and potential of overdiagnosing. It was also mentioned that, for the DSM-5, the idea was indeed to slim it down, and reduce the number of diagnoses (Table 5, quote 16). Other disadvantages being reported were that it may create false positives, and brand people as sick when they are actually not. It was noted that it might also draw focus from the cause of the problem, and that, as opposed to people who benefit from a label for what they suffer from, some people find a diagnosis to do more harm than good (Table 5, quotes 17 and 18).

### Diagnostic C

#### Questionnaire

Among different aspects of the diagnostic criteria, exercise related symptoms were specifically covered in the questionnaire. A majority of the participants reported thinking that these exercise related symptoms should be part of the diagnostic criteria for ON (*p* < 0.001). Associations between respondent characteristics and their opinion on whether exercise related symptoms should be part of the diagnostic criteria were also examined, but no significant associations were found.

#### Interviews

Although some parts of the diagnostic criteria were considered suitable by the interview participants, most agreed that the criteria needed to be refined. The main refinement mentioned was to make the criteria clearer and more specific. A concrete suggestion to improve this was to have more examples (Table 5, quote 19). It was also mentioned that the medical complications ought to be more specific (Table 5, quote 20). No specific comments were made on exercise related symptoms to be included in the diagnostic criteria.

## Discussion

Although some previous researchers have deliberated on a potential link between ON and other disorders, no empirical studies have been conducted to investigate the opinions of clinically active health professionals on the matter, making our study the first of its kind. This is an important step to take in the early stages of research on the condition, and it was an attempt by the co-authors to acquire some clarity and direction on the topic. It was clear that participants in this study recognized ON, reported that they believed it to be prevalent in the Netherlands and reported having met clients who fulfilled the proposed diagnostic criteria. Furthermore, the majority of participants agreed that the condition should have its own diagnosis, placed in the DSM category Eating and Feeding Disorders.

Questionnaire results revealed that PHP were more in favor of a separate diagnosis than were MHP. A possible explanation for this may be the different clientele and focus of the profession groups, as PHP work more with the body and MHP more with the mind. Existing literature suggests that individuals working with the body professionally may be predisposed to ON, as prevalence among dietitians has been estimated to 41.9% (Asil and Sürücüoğlu, 2015), possibly making them more alert to these kinds of symptoms and their presentation in their patients. The results of the current study also show that PHP had met clients who fulfilled the proposed criteria for ON to a greater extent than MHP. From an external view, it can look like someone with ON is healthy, and the person may not admit (to themselves or their environment) that they have a problem. Therefore, an individual with ON may not seek care so easily. They might, however, seek support in “healthy” habits for eating and exercising, thus bringing them into contact with dietitians or physiotherapists earlier and more frequently than psychologists or psychiatrists. This displays the importance of including a wide range of health professionals in a study like this, and may have led to a more pressing sense of urgency regarding a diagnosis for the condition among PHP. It is, however, important to remember that PHP usually do not have specific training in mental health diagnoses.

An important question in this discussion is to what extent a diagnosis will be beneficial for the patient. On the one hand, patients will benefit from the recognition of ON as a distinct disorder, as it may lead to improved quality of treatment as well as potentially improving access to treatment should insurance companies decide to view it as a condition for which reimbursement can be provided. On the other hand, there is always a risk of stigmatization of individuals with a distinct condition, calling for caution in regard to the decision. Regardless of the potential advantages or disadvantages of a diagnosis, one may argue that a condition that is pathological and differs from currently available diagnoses should be represented in the DSM. The diagnoses of the DSM should be representative of reality; thus, if it is occurring in real life as a condition, it should have a place in the DSM.

Regardless of decisions regarding diagnosis, the level of impairment caused by the condition needs to be central in the treatment of the condition. With this in mind, it is essential to talk to patients and to know what they experience. Patient experiences are central in formulating good and valid diagnostic criteria, however the diagnostic criteria are needed to identify study participants. This discrepancy may pose a problem with identification of study participants.

Besides determining whether ON should have its own diagnosis at all, there is also the question of where this potential new diagnosis should be documented. In this study we focused on the DSM, however, many respondents expressed mixed opinions regarding this manual and mentioned several shortcomings. As diagnoses are fluid and constantly changing, the DSM is regularly updated. Although it is positive that the manual attempts to stay up to date with current situations, the processes regarding the development of the DSM and whether the updates fulfill their purposes have previously been criticized (Caplan, 1991; Möller et al., 2015).

The importance of conducting a study like this one lies largely in that it raises awareness in the research community as well as among clinicians. It gives insight into the current state of knowledge and beliefs among practicing health professionals, while also letting them know that their experiences and opinions are valued. The mixed-methods design allowed for identification of a broad range of opinions of some health professionals in the Netherlands on whether, and how, ON should be classified, as well as their reasoning behind the opinions.

A large limitation to this study is the representativeness of the sample and thus generalizability of the results. A total N of 1165 clinicians were directly contacted and invited to participate in the study, however, only 13.7% of these decided to do so. With such a small proportion participating, the representativeness of the sample may be jeopardized, as it may differ systematically from the Dutch clinicians in these professions. It is also quite possible that only individuals with a specific interest in this topic participated, which may have influenced the results of this study further. Additionally, as the inclusion criteria were broad, the participants’ pool was a rather diverse yet small group. Although this means that the sample is not representative of all different kinds of health professionals in the Netherlands, and less so globally, it does protect against “tunnel vision” in these early stages of exploring the condition. As it is not yet known how ON should be categorized, and we do not even know whether it indeed is an eating disorder, only including participants with extensive experience with eating disorders may lead to biased results. For generalizability and to assure representativeness, this study would need to be replicated. After having conducted this study it is clear that this design is feasible, therefore, the study also serves as a feasibility study.

Additional limitations to this study were the use of external assistance with coding of the interviews as well as the validity of the questionnaire. Due to language barriers, external assistance was necessary to code the interviews, possibly resulting in nuances being lost in the analysis. To minimize this, a close and regular contact was held between FR and the coding assistant during this process, ensuring that no questions were left unanswered. Regarding the validity of the questionnaire, no specific tests were conducted for this. Pre-designed testing of construct validity could have enhanced the quality on this matter.

Mental disorders, such as AN and OCD, have been proven to result in tremendous suffering for the individual, as well as an increased risk of suicide. ON seems to have many similarities with these disorders, while also possibly reaching prevalence numbers several times higher than those of AN (American Psychiatric Association, 2013; Dunn and Bratman, 2016). This makes further research imperative. The findings of this study suggest that some health professionals in the Netherlands may believe that ON should have a separate diagnosis and report that they recognize it as prevalent in their work as well as their everyday lives. However, in order to permit further generalization this study needs to be replicated in different settings and with larger samples. Investigations of how individuals with these symptoms experience it, what impairment it causes and further refinement of the criteria are also crucial. This must then be followed by more robust and valid studies on prevalence.

## Conclusion

In conclusion, a heterogeneous sample of health professionals in the Netherlands seem to be of the opinion that ON should have its own diagnosis and that it should be placed in the DSM category Eating and Feeding Disorders. The results of this study suggest that, despite some possible disadvantages, giving ON its own diagnosis might be helpful to both patients and health professionals by improving treatment and facilitating the therapy process. However, as the sample cannot be considered to be representative, more research is needed to allow for further generalization.

## Data Availability

The raw data supporting the conclusions of this manuscript will be made available by the authors, without undue reservation, to any qualified researcher.

## Author Contributions

FR participated in all parts of the research, conceiving the idea, design and conceptualization of the study, collecting and analyzing the data and writing a first draft of the manuscript. ES participated in conceiving the idea and supervised and guided all parts of the research as well as participated in preparing the manuscript for publication. ZB participated in the design of the study and collecting the data. TC participated in the data analysis and in preparing the manuscript for publication. All authors discussed the results and contributed to the final manuscript.

## Conflict of Interest Statement

The authors declare that the research was conducted in the absence of any commercial or financial relationships that could be construed as a potential conflict of interest.
