# Supplementary material for: Orthorexia Nervosa: Disorder or Not? Opinions of Dutch Health Professionals
Source: Front Psychol. 2019 Mar 15;10:555. doi: 10.3389/fpsyg.2019.00555 (PMC6428718; doi:10.3389/fpsyg.2019.00555)
Supplement: Supplementary file 1 [file Data_Sheet_1.docx]

Supplementary Material

Orthorexia Nervosa: disorder or not? Opinions of Dutch health professionals

**Frida V. M. Ryman*, Tomris Cesuroglu, Zarah M. Bood, Elena V. Syurina**

***Correspondence:** Frida V. M. Ryman: frida.ryman@gmail.com

**APPENDIX A. FRAMEWORK**

**
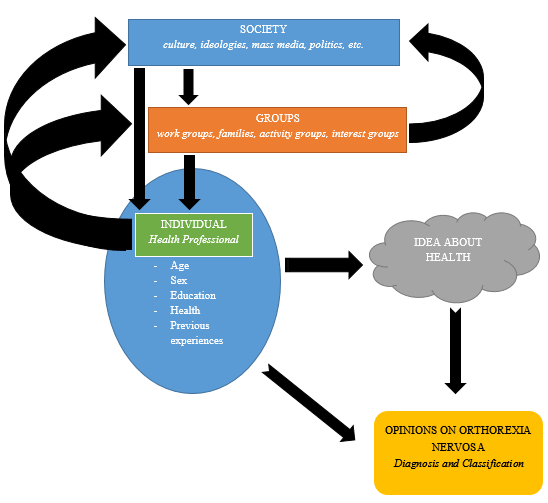
**

**APPENDIX B. QUESTIONNAIRE**

**Introduction**

This questionnaire is part of a research project about Orthorexia Nervosa. Orthorexia Nervosa is an emerging disordered eating pattern, in which the individual acquires a pathological obsession with healthy eating. This can have physical, psychological and social consequences. As it is a relatively new condition, little research has been conducted on it. There is no consensus regarding diagnostic criteria, and the studies that have been conducted on it are inconclusive.

The aim of this study is to shed light on the perspectives of health professionals on the condition, current diagnostic criteria, possible influential factors and how Orthorexia Nervosa should be classified. The required data is collected through interviews and this questionnaire.

The questionnaire will consist of three chapters:

- Chapter 1: Recognition, diagnosis, and classification of Orthorexia.

- Chapter 2: Influence modern Western culture on Orthorexia.

- Chapter 3: Characteristics of respondent.

Overall, it will take approximately 10 min to fill in the questionnaire. All your answers will stay anonymous. If you would like to receive the results of the research, you can send an email to [orthorexiaresearch@gmail.com](mailto:orthorexiaresearch@gmail.com). In this way, your email cannot be linked to your response on the questionnaire.

**Chapter 1: Recognition, Diagnosis, and Classification of Orthorexia**

**Recognition and Diagnosis**

This chapter consists of questions regarding recognition, diagnosis and classification of Orthorexia Nervosa.

*- > Picture of criteria of ON here*

Have you met clients who fulfill these criteria?

1. Yes, within the last year

2. Yes, more than 1 year ago

3. No

How prevalent do you the think the condition is in the general population in the Netherlands (1 meaning not at all prevalent and 5 meaning extremely prevalent)?

1 2 3 4 5

Do you think Orthorexia Nervosa should have its own diagnosis in the upcoming versions of the DSM?

Yes/No

*If no*, which existing diagnosis do you think it fits within? *Multiple options are possible*

1. Anorexia Nervosa

2. Bulimia Nervosa

3. Avoidant/Restrictive Food Intake Disorder (ARFID)

4. Obsessive and Compulsive Disorder

5. General Anxiety Disorder

6. Other:_____________

**Classification**

In the DSM-5, diagnoses are divided into different categories of diseases or disorders of the same type.

Which diagnostic category or categories does Orthorexia Nervosa fall under according to your opinion?

*Multiple options are possible*

1. Eating and Feeding Disorders

2. Obsessive Compulsive Disorders

3. Anxiety Disorders

4. Other: ______________

**Additional Factors**

In the media, several factors are presented as possible contributors or part of the condition.

On a scale from 1 to 5, to what extent do you think each of the following factors contribute to/are part of Orthorexia Nervosa (1 meaning not a contributor/not part of the condition at all and 5 meaning a vital contributor/great part of the condition)?

• Exercise 1 2 3 4 5

• Weight loss 1 2 3 4 5

Do you think exercise related symptoms should be part of the diagnostic criteria?

Yes/No

**Chapter 2: Influence Modern Western Culture on Orthorexia**

In this chapter you will get questions regarding a possible influence of the modern Western culture on the emergence of Orthorexia. This questionnaire will not ask about other factors that could influence orthorexia, but the fact that they are prevalent is acknowledged.

On scale of 1–5, with 1 meaning no influence at all and 5 meaning a great influence, to what extent do you consider each of the following parts of the modern Western culture to influence the emergence of Orthorexia?

*Worldviews and values in the modern Western culture:*

• Individualism 1 2 3 4 5

• Materialism 1 2 3 4 5

• Capitalism 1 2 3 4 5

*Profit-seeking market and its industries:*

• Food industry 1 2 3 4 5

• Diet- and weight loss industry 1 2 3 4 5

• Fitness industry 1 2 3 4 5

• Fashion industry 1 2 3 4 5

• Cosmetic surgery industry 1 2 3 4 5

*Mass media:*

• Broadcast media (Television, movies, and radio) 1 2 3 4 5

• Digital media (Internet) 1 2 3 4 5

• Printed media (Books, newspapers, and magazines) 1 2 3 4 5

• Outdoor advertisements (Billboards, shops, etc.) 1 2 3 4 5

The worldviews, values, industries, and the mass media prevalent in the modern Western culture, shape perceptions and behaviors of individuals. On scale of 1–5, with 1 meaning no influence at all and 5 meaning a great influence, to what extent do you consider the following perceptions and behaviors within the modern Western culture to influence the emergence of Orthorexia?

*Beauty ideals within the modern Western culture:*

• Thin body ideal 1 2 3 4 5

• Muscular body ideal 1 2 3 4 5

*Perceptions within the modern Western culture on what is (un)healthy:*

• Fast food is unhealthy 1 2 3 4 5

• Biological/organic/vegan food is the healthiest 1 2 3 4 5

• Low fat/low carb/gluten free food is the healthiest 1 2 3 4 5

• Regular exercise is best for the body 1 2 3 4 5

*Eating behaviors within the modern Western culture:*

• Eating fast food 1 2 3 4 5

• Trends of having healthy diets (organic, low carb, etc.) 1 2 3 4 5

**Chapter 3: Characteristics of Respondent**

This chapter consists of questions regarding your characteristics.

Gender

1. Male

2. Female

3. Not listed: ________

4. Prefer not to answer

Age _______________________

Ethnicity

1. Caucasian

2. Black or African American

3. Asian

4. Native Hawaiian or Pacific Islander

5. Other: ___________

Highest level of education

1. HBO

2. Bachelor’s Degree

3. Master’s Degree

4. Other: ___________

What is your profession?

1. Psychologist

2. Psychiatrist

3. Dietitian

4. Physiotherapist

For how many years have you been practicing this profession? _______________________

What languages do you treat patients in?

*Multiple options possible.*

1. Dutch

2. English

3. Spanish

4. French

5. Italian

6. Other: ____________

Have you encountered patient(s) with a suspected or confirmed eating disorder in the clinic? Yes/No

End

Do you have anything you would like to add or comment on?

_____________________________________________________________

_____________________________________________________________

_____________________________________________________________

_____________________________________________________________

Do you have any advice or comments regarding the questionnaire itself?

_____________________________________________________________

_____________________________________________________________

_____________________________________________________________

_____________________________________________________________

If you would like to receive the results of this study or if you have any questions, you can send an email to [*orthorexiaresearch@gmail.com*](mailto:orthorexiaresearch@gmail.com).

Thank you for your participation!

**APPENDIX C. INTERVIEW GUIDE**

**Introduction**

Welcome (name participant),

First, I would like to thank you for your participation in this study and for allowing us to have this interview with you. My name is (name interviewer) and I am conducting this interview as part of a study about professional’s opinions regarding a newly emerging eating pattern called orthorexia nervosa. The other researchers involved in this study are Elena Syurina, main researcher on the project and assistant professor at the VU and Maastricht University, and Zoë Bood and Alice Geurtsen.

The objective of the research project is to gain insight into the perspectives of health professionals in the Netherlands regarding Orthorexia Nervosa. Individuals with Orthorexia Nervosa acquire a pathological obsession with healthy eating. Even though it is frequently mentioned in media, little research has been conducted on it. Therefore, there is no consensus regarding diagnostic criteria, and the studies that have been conducted on it are inconclusive. By means of interviews and a questionnaire this study will explore how health professionals in the Netherlands view the condition, its diagnostic criteria, factors that influence it and how it should be classified. This will result in improved knowledge and understanding of the condition and facilitate the development of treatments for it, leading to better clinical care for patients.

As for this interview, feel free to ask questions whenever you want, and take as much time as you need to answer the questions. You are not obligated to answer a question if you do not feel comfortable to do so. Participation is voluntary and you may withdraw from the study at any given time without giving reasons. Please note that we handle the information provided to us with the utmost care. All your data will be anonymized and stored at a safe and password protected database. All personally identifiable information will be deleted during the analysis of the data, so that no one can trace your answers back to you. Also, during the interview I will provide you with an infographic with information about a fictional patient, with which I would like to work interactively with you. You will be provided with more information about this later in the interview. Finally, I would like to ask for your permission to record this interview and to analyze the answers provided for the purposes of this study.

Now that you know more about the research, are you still willing to participate in the study? [if yes] Would you be so kind to sign the informed consent form?

**[Provide participant with informed consent form]**

If it is alright with you, I would like to turn on the recorder now?

**[Turn recorder on if allowed, otherwise, make sure to have pens and paper at hand]**

Now, on record, is it okay if we record and use the data from this interview for the purposes of this project?

# Background information interviewee

I would like to start off by asking you some questions about yourself. This data will be used as background information during the analysis of the results.

- How old are you?
- Where are you from?
- For how long have you been working as a psychologist/psychiatrist?
- Where did you go to school?
  - What type of study program did you follow?
  - Did you take any additional courses/certificates that could be of interest for this study *[e.g. a course about eating disorders]?*
- What kind of practice/clinic are you working in?
  - What types of patients do you see there *[e.g. children, veterans, immigrants, OCD patients, inpatient/outpatient]?*
- What experience do you have with patients with eating disorders?
- What types of eating disorders did you encounter during your work as a psychologist/psychiatrist *[e.g. anorexia, bulimia, binge eating disorder]?*
- How many patients (approximately) did you treat for having an eating disorder during the past *year/month/week*?
- What other types of conditions do you see in your work?
- Do you see individuals with OCD, personality disorders, and anxiety disorders?
- How many patients (approximately) did you treat for having OCD/personality disorders/anxiety disorders during the past *year/month/week*?

# Diagnosis and classification

I will now show you a picture of a hypothetical patient who might come into your clinic and I will ask you some questions about this patient.

**[Provide participant with picture and markers: take the participant through the picture so that you are sure that he/she understands it completely, also explain that he/she can fill in possible diagnosis in the box at the center of the picture]**

Let us say that a patient presents with these symptoms.

- Who do you see in front of you? Do you imagine this to be a specific patient? How would you describe the characteristics/personality of this patient? There are no right or wrong answers, I would just like to know what you think.
  - *(Age, gender, ethnicity, education etc)*
- Are you able to diagnose this person based on current classifications, such as the DSM 5?
- ***If yes* →** How would you diagnose this patient?
- Why do you think it should be diagnosed like this? Why shouldn’t it be diagnosed as something else (*e.g. if they state that it is bulimia, why not anorexia)?*
- Is the diagnosis sufficient? If yes, why and if no, why not?
- Do you see these kinds of patients in your work? How often?
- ***If no* →** Why not? What else would you need to know?

Currently, these are the proposed diagnostic criteria of orthorexia nervosa

**[Show a table with the criteria from Dunn and Bratman. Go through them with the participant].**

- So now that you have been presented with another possible diagnosis, would you change the diagnosis of the hypothetical patient or do you still feel that the diagnosis you were able to give according to current classifications is more fitting? Why?
- ***If the original diagnosis fits better* →** Does that mean that you do not think the condition should have its own diagnosis?
  - Why/why not?
  - Advantages/disadvantages?
- Do you think that a separate diagnosis for ON would contribute to improved treatment possibilities for patients?
  - Do you think that this is important?
  - Why/why not?
- Even though you do not think this fictional patient fit the criteria for orthorexia, do you see patients who you believe do? If yes, how often?
- ***If the orthorexia diagnosis fits better* →** Does that mean that you think it should have its own diagnosis?
  - Why/why not?
  - Advantages/disadvantages?
- Do you think that a separate diagnosis for ON would contribute to improved treatment possibilities for patients?
  - Do you think that this is important?
  - Why/why not?
- Do you see patients who you believe do fit the criteria for orthorexia? If yes, how often?

**[Show the participant the list of possible diagnostic categories in the DSM-V]**

- Looking at the current diagnostic categories in the DSM 5, where do you think orthorexia fits in? In what category/categories does it belong? **(Multiple possibilities and no wrong answers).**
- ***If more than one category is mentioned →***
  - Could you explain why you think it fits in multiple categories?
  - If you had to choose one, where do you think it would fit best?

Influence modern Western culture

Now I would like to talk to you about another aspect of orthorexia, namely the link between orthorexia and culture.

Orthorexia is mainly found to be prevalent in developed countries, meaning that there COULD be a link between the Western culture and the emergence of orthorexia. However, no research has yet been conducted on this. I would like to discuss the possibility of this link with you.

- What do you perceive as part of the modern Western culture?
  **[If the participant is stuck or does not have a sufficient idea about it, you can help them with this: *Modern Western culture could e.g. consist of:
  Worldviews and values like individualism, materialism, capitalism
  Profit seeking industries; food, diet- and weight-loss, and fitness industries
  Mass media (used by the industries))***
- What are your thoughts on the link between the modern Western culture and orthorexia?
- Do you think that the modern Western culture influences the emergence of orthorexia?
- ***If suggested that there is a link* 🡪**
- Why do you think that?
- How does the modern Western Culture influence the emergence of orthorexia? In what ways? **(You can think about influencing eating behaviors, body image ideals, perceptions of health/illness. Do not provide them with this unless they really have nothing to work with.)**
- To what degree does the modern Western culture influence the emergence of ON? Is there a strong or minor influence?
- What about other cultures; do you think other cultures may be linked to orthorexia or it is only the Western culture?
- ***If not suggested that there is a link 🡪***
- Why not? Can you elaborate?
- How about other cultures; do you think other cultures may be linked to orthorexia?

# End

That was the last question I had for now. Is there anything else you would like to add?

To finish off, I would, again, like to ask you if it is okay with you that the data from this interview is used within the study?

Finally, I would like to thank you for taking the time to do this interview. If you would like, I can send you the article when it is finished. You can also get a summary or transcript of the interview if you want.

**APPENDIX D. INFOGRAPHIC: HYPOTHETICAL CASE**

**
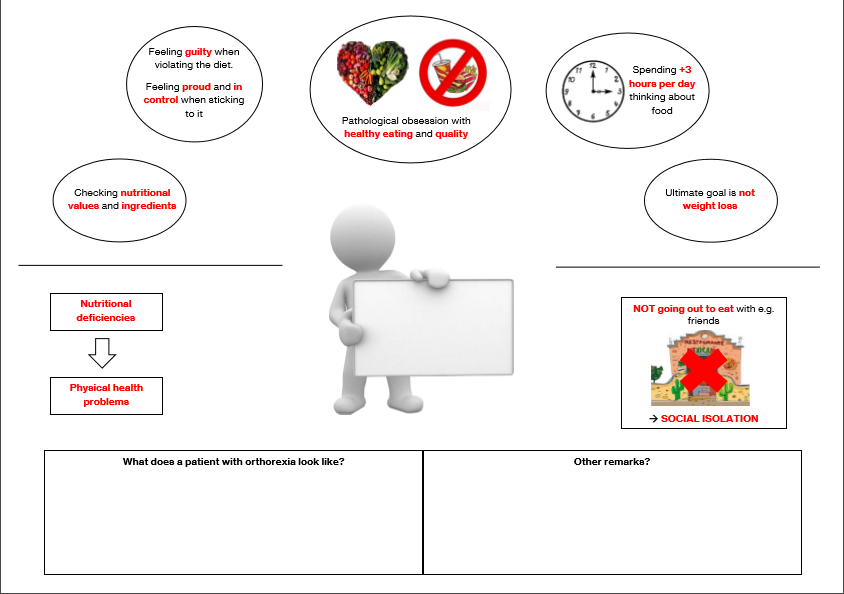
**

**APPENDIX E. CODE BOOK**

1. **DIAGNOSIS AND CLASSIFICATION**
   1. **Idea of prevalence of ON**
      1. Belief that ON is prevalent
      2. Belief that ON is not prevalent
   2. **Thoughts on comorbidity**
      1. Comorbidity is common
      2. Comorbidity is not common
   3. **Case**
      1. Patient characteristics
         1. *Gender*
            1. *Female*
            2. *Male*
            3. *No specific gender*
         2. *Age*
            1. *Younger*
            2. *Older*
            3. *No specific age*
         3. *Ethnicity*
         4. *Personality traits*
            1. *Need for control*
            2. *Aware*
            3. *Rigid (personality/mind)*
            4. *Sporty*
         5. *Appearance*
         6. *Education*
            1. *Higher education (bachelor/HBO and higher)*
            2. *Lower education (below bachelors/HBO)*
         7. *Occupation*
         8. *History*
            1. *Previous eating disorder (AN or BN)*
            2. *Previous lack of control (in a specific event/situation or such)*
      2. Diagnosis
         1. *Based on current classification*
         2. *Preferred diagnosis*
            1. *Currently available diagnosis rather than ON*
            2. *ON rather than currently available diagnosis*
   4. **Thoughts on giving ON its own diagnosis**
      1. Hesitant to the helpfulness of diagnoses in general
      2. Negative to own diagnosis
      3. Positive to own diagnosis
      4. Advantages to giving ON its own diagnosis
         1. *Nuancing*
         2. *Awareness*
         3. *Facilitate coping for patients*
         4. *Improve treatment*
         5. *Facilitate health professionals’ work with these patients*
         6. *Insurance refunds*
         7. *DSM up-to-date with societal changes*
      5. Disadvantages to giving ON its own diagnosis
         1. *Too much diagnostics*
         2. *Draws focus from the root cause of the problem*
         3. *Diagnoses do more harm than good to some people*
         4. *May create false positives*
      6. Need for research
   5. **Contextualization**
      1. Mental conditions present differently in different contexts
      2. Diagnoses cannot be applied globally
   6. **Thoughts on current proposed criteria**
      1. Need for refined criteria
         1. *Parts that need refinement*
         2. *Positive parts of the criteria*
      2. Categorization
         1. *Possible categories*
         2. *Preferred category*
